# Identification, rearing, and distribution of stick insects of Madeira Island: An example of raising biodiversity awareness

**DOI:** 10.1093/jis/14.1.49

**Published:** 2014-01-01

**Authors:** António M. F. Aguiar, Dora Aguin Pombo, Ysabel M. Gonçalves

**Affiliations:** 1 Secretaria Regional do Ambiente e Recursos Naturais, Laboratório de Qualidade Agrícola, Caminho Municipal dos Caboucos, 61, 9135-372, Camacha, Madeira, Portugal; 2 Universidade da Madeira, 9000-390 Funchal, Madeira, Portugal; 3 CIBIO, Centro de Investigação em Biodiversidade e Recursos Genéticos, Universidade do Porto, 4485-601 Vairão, Portugal; 4 Museu de História Natural do Funchal, Rua da Mouraria, 31, 9004-546 Funchal, Madeira, Portugal

**Keywords:** Bacillidae, *Clonopsis gallica*, *Carausius morosus*, Macaronesia, Phasmatidae

## Abstract

Two species of stick insects are currently known to be present in the Macaronesian archipelagos: *Clonopsis gallica* (Charpentier) (Phasmatodea: Bacillidae) on the Canary Islands and in the Azores and *Carausius morosus* (Sinéty) (Phasmatidae) in the Azores. Here, we provide the first reliable records of the presence and distribution of *C. gallica* and *C. morosus* on Madeira Island. Egg and adult stages are briefly described along with some notes on the life history of these species in captivity. Data on islandwide distribution are based on specimens donated by the public in response to an article published in a daily newspaper. This method of data collection raised great popular interest in stick insects. The role of newspapers as a means of communicating awareness in biodiversity issues is discussed.

## Introduction

The apparent paucity of stick insect (Phasmatodea) species in Macaronesia is surprising when compared with the stick insect faunas of other islands. A recent checklist of terrestrial taxa from Madeira and Selvagens archipelagos lists 3,394 taxa of arthropods (3,097 are insects), of which about 30% are endemic ( Borges et al. 2008 ). However, this list does not provide records of Phasmatodea. The absence of stick insect records from Madeira was referred to by Baéz (1993) , who considered the single specimen observed by M. Biscoito, the director of the Museum of Natural History of Funchal, a likely case of ‘fortuitous introduction.’ According to van Harten (1993) and Arechavaleta et al. (2005) , records of Phasmatodea from the Cape Verde archipelago are also unknown. However, the Canary Islands archipelago has one confirmed species, *Clonopsis gallica* (Charpentier) (Bacillidae) *,* and the Azores has this species and *Carausius morosus* (Sinéty) (Phasmatidae). Both species are considered to be non-native to these archipelagos ( Lopéz and Morales 2010 ; Sousa 2010 ).

More specifically, *C. gallica* has being reported from Tenerife (Canary Islands) and from Faial and São Miguel (Azores). In the Canary Islands, this species was first cited by Bolívar (1926) , who didn’t give an indication of a specific island, and afterward by Chopard (1954) . In the Azores, it was first cited by Bolívar (1894) from São Miguel Island and later from Faial Island by Chopard (1940) . More recently, Baéz (1996) collected a few specimens on Tenerife in a cultivated area to the north side of the island. In addition, there are some other unreliable records of an un-documented species for this archipelago. The identification of some of these species is questionable, and their presence still remains unstudied ( Blands et al. 1996 ). In the Azores, *C. morosus* has been reported for Faial and Terceira by Sousa (2010) and more recently from Santa Maria and São Miguel islands by Borges et al. (2013) .

Species of stick insects range in size from large to very large, with one recently described Malaysian species, *Phobaeticus chani* Bragg, having an astounding total length of 570 mm. This is considered the largest extant insect known to date ( Hennemann and Conle 2008 ). However, despite their large size, stick insects have very effective camouflage, adopting the appearance of sticks or leaves. Together with low population densities, this may be one of the reasons why so few specimens have been collected until recently in Macaronesian archipelagos. Over the last decade, stick insects have occasionally been found by professional entomologists, and recently many people have started to find them in houses across the Madeira Island. This clearly suggests that the populations are increasing in density and distribution. As a result, interest has been sparked among the general public and requests for information were made to a local newspaper.

In response to this sudden interest, the authors were contacted by the local newspaper for more information such as: how many species live on Madeira, when did they first appear, whether they were originally from Madeira, and if they could cause damage to crops or people. Our interest was raised and, as we did not feel qualified to answer many of the questions, we made a request through the paper inviting people to bring us specimens. There was an excellent response to our request which allowed us to generate a very comprehensive distribution database. Collectors were pleased to know that their name could appear on the publication of this information. This sudden interest leads us to publish these records and reflect on communicating biodiversity awareness: are we doing the right things to generate interest in people and convince them that biodiversity and taxonomy are important?

## Materials and Methods

The classification adopted follows the Phasmida Species File online database by Paul Brock ( http://Phasmida.SpeciesFile.org ). The specimens studied and collected by the general public were deposited in the following Institutions:

MMF – Museu Municipal do Funchal (História Natural), Funchal, Madeira, Portugal ICLAM – Laboratório de Qualidade Agrícola, Camacha, Madeira, Portugal UMa – Universidade da Madeira, Funchal, Madeira, Portugal

### Insect sampling

Madeira is an oceanic island located in the North Atlantic at about 635 km from North Africa (Morocco) and 849 km from Europe (Sagres, Portugal). In Madeira, most specimens of *Clonopsis gallica* and *Carasius morosus* were sampled by citizens primarily in houses and gardens by hand collecting, although some specimens were collected during regular entomological field trips by two of the authors (A. Aguiar and Y. Gonçalves).

### Insect rearing

A female *Clonopsis gallica* only a few days old was collected on 15 April 2010 and an adult mature female *Carasius morosus* was collected on 28 June 2010 in the same locality: Assomada, Madeira, 28SCB2914 (UTM). They were fed in the laboratory on elm leaf blackberry, *Rubus ulmifolius* Schott (Rosales: Rosaceae), until they died eight months later.The stick insects were kept in round plastic breeding cages with leafy branches of *R. ulmifolius* provided in jars of water that were replenished each week. The cages consisted of transparent cylinders 20 cm in diameter and 40 cm high, with a base and lid of durable green polythene. Throughout their development, in an unheated room, no particular light source was used other than the room artificial illumination consisting of eight fluorescent tubes, which were turned on for at least seven hours each day. Eggs were collected from the floor of the cages at regular periods and kept in separate cages. The preoviposition, oviposition, and postoviposition periods were registered. The preoviposition period is considered the number of days required for females to start laying eggs following eclosion, the ovipositon period refers to the number of days they oviposit, and the postoviposition period is equivalent to the number of days they survive after oviposition has terminated.

## Results

### Identification of species breeding in Madeira

**Figure 1.**
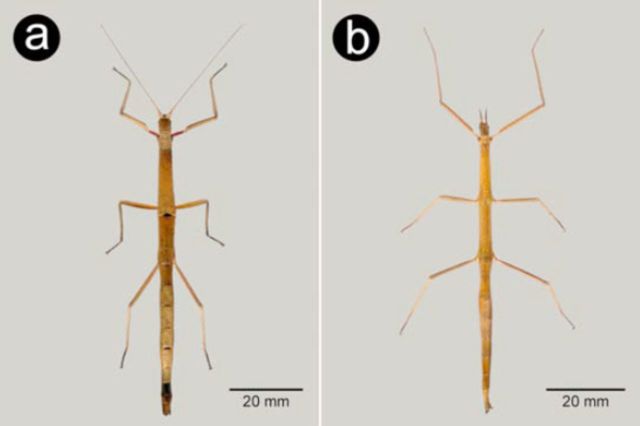
General habitus in dorsal view: a – female *Carausius morosus* , b – female *Clonopsis gallica* . High quality figures are available online.

**Figure 2.**
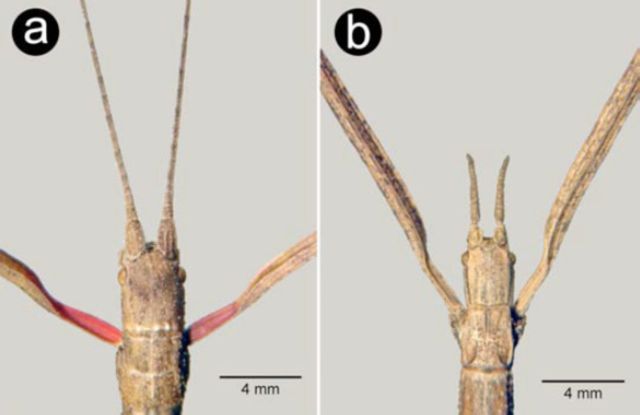
Details of head, antennae, and pronotum in dorsal view: a – female *Carausius morosus* , b – female *Clonopsis gallica* . High quality figures are available online.

**Figure 3.**
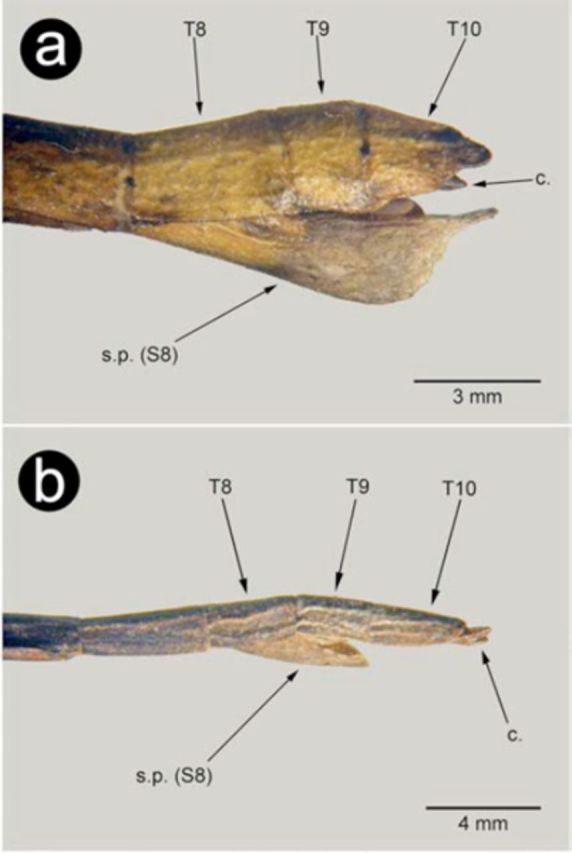
Details of last abdominal segments of female: a – *Carausius morosus* , b – *Clonopsis gallica* . T – tergites, S – sternite, c – cerci, d.v. – dorsal valvulae, s.p. – subgenital plate. High quality figures are available online.

**Figure 4.**
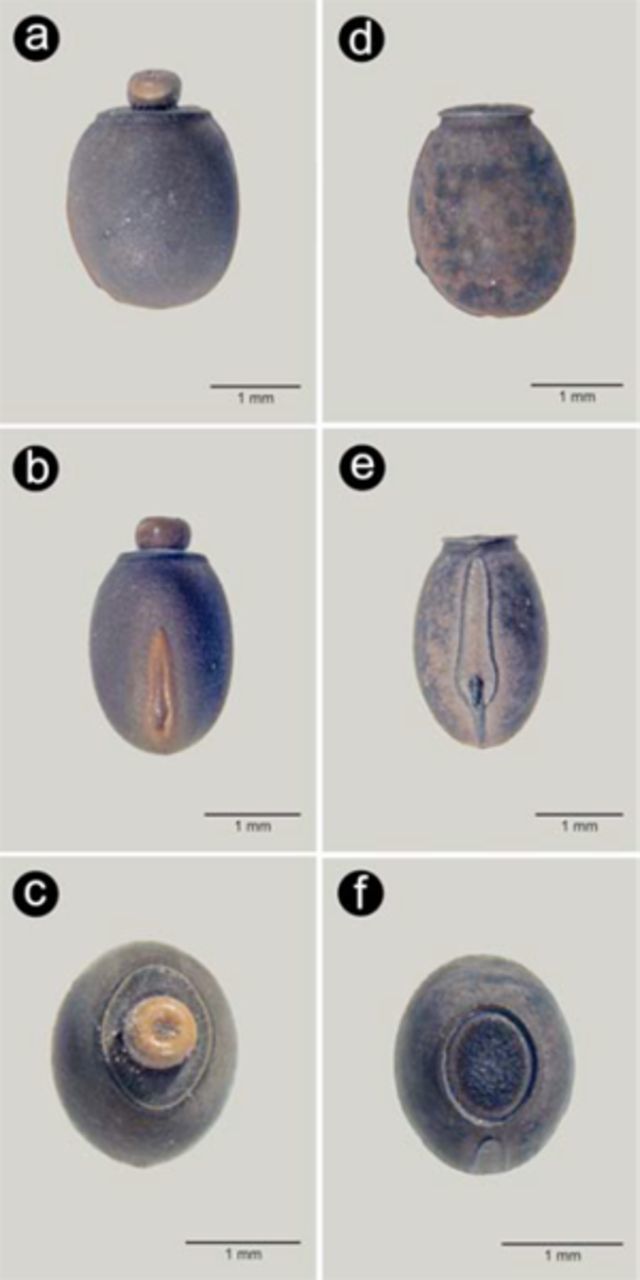
Comparison of both species eggs: lateral view (a, d), dorsal view (b, e), anterior pole (c, f); a, b, c – *Carausius morosus* , d, e, f – *Clonopsis gallica* . High quality figures are available online.

The two species of stick insect, *Clonopsis gallica* and *Carausius morosus* , presently breeding in the wild on Madeira Island can be easily differentiated macroscopically. These stick insects are parthenogenetic, and so, as males are extremely rare in nature, we will be referring exclusively to female characters (see Table 1 ). The most common species, *C. morosus* , has a slightly longer body ( Figure 1a ) and long antennae ( Figure 1a , 2a ), almost filiform with numerous small segments; the inner face of the fore femur’s base is bright red ( Figure 2a ); the subgenital plate reaches the apex of tergite 10 ( Figure 3a ); the eggs are globose, brown, and the operculum located at the anterior pole has a button-like yellowish capitulum ( Figure 4a , b , c ) and a brownish micropylar plate occupying about half length of the egg’s dorsal surface. The second species, *C. gallica* , has short antennae with 13 segments ( Figure 1b , 2b ); inner face of fore femur’s base the same color as body ( Figure 2b ); subgenital plate reaches the apex of tergite 9 ( Figure 3b ), and the egg of similar shape but the operculum does not present a capitulum and the micropylar plate has the same colour of the main capsule, occupying about 0.66 of the egg’s length ( Figure 4d , e , f ).

**Table 1.**
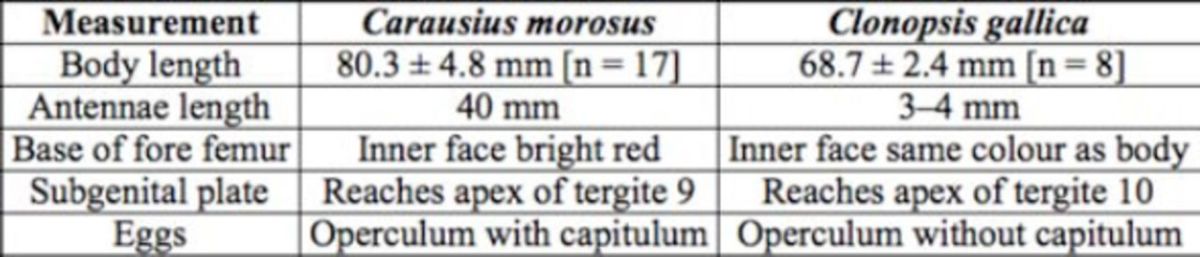
Main distinguishing characters differentiating the females of both species.

Infraorder **Anareolatae**

Family **Phasmatidae**

****Carausius morosus**** ( Sinéty 1901 )

*Dixippus morosus,* Sinéty 1901 , Cellula 19: 121 [Type locality: Asia-Tropical, Indian Subcontinent, India, Shambaganur]

**Material studied** (36 females, 3 nymphs): MMF 27694 – 1F, Rua da Rochinha, Funchal, 28SCB2213, 2.iv.1990, Rui Humberto Correia Luiz leg.; UMa – 1 nymph; Palheiro Ferreiro, 28SCB2414, 10.i.2002, José Jesus leg.; Uma – 2F, Port of Funchal, 28SCB2012, 30.x.2002, Isabel leg.; MMF 36293 – 1F, Caminho da Achada, Funchal, 28SCB2014, ii.2003, Miguel S. Gonçalves leg.; MMF 36289 – 1F, Livramento, Funchal, 28SCB2115, 14.xii.2003, Roberto Mendes leg.; UMa – 1F, Ribeira de São Gonçalo, Funchal, 28SCB2313, 2.iv.2005, Mario Correia leg.; MMF 36291 (a,b) – 2F, Longueira, Campanário, 28SCB1016, 31.viii.2005, J. Luis Sousa leg.; MMF 36292 – 1F, Longueira, Campanário, 28SCB1016, 26.ix.2005, Ana Luisa Sousa leg.; UMa – 1 nymph, Centre of Funchal city, 35 m, 28SCB2113, 11.xi.2005, Márcio Nóbrega leg.; MMF 36617 – 1F, S. Gonçalo, Funchal, 28SCB2414, 24.vi.2006, Maria R. Gonçalves leg. (laid 47 eggs in captivity); UMa – 1F, Monte, Corujeira, 28SCB2116, 30.vi.2006, Marcio Nóbrega leg., on *Acacia* sp.; ICLAM 02905 – 16F, Palheiro Ferreiro, Urb. de São Gonçalo, Funchal, ex. *Lavandula angustifolia* 28SCB2414, 446 m, 7.iii.2007; UMa – 1F, Cancela 7.iii.2008, Henrique Rosa leg., (laid 20 eggs in captivity); UMa – 1F, Monte, Funchal, 28SCB2216, 16.xii.2009, Maria Jesus Viveiros leg.; UMa – 1F, Santo António, Lombo dos Aguiares, 28SCB1816, 8.iii.2010, Nelson Aguiar leg.; UMa – 2 F, Caminho do Lombo, Monte, Funchal, ±340m, 28SCB2215, 27.iii.2010, Fabio Reis leg.; UMa – 1 nymph and photo of an adult taken on 1.v.2010 by Célia Fernández; UMa – 1F, Madeira, date and collector unknown; ICLAM 03237 – 1F, Assomada, Caniço, Santa Cruz, 28SCB2914, 28.vi.2010, Lina Noite leg.; ICLAM 03241 – 1F, São Vicente, village centre, 40 m, 28SCB0830, 20.Jan.2011, Joel Freitas leg.; UMa – 1F, Caminho do Lombo, Monte, ±340m, 28SCB2215, 9.iv.2011, Fabio Reis Leg.

This species seems to be largely distributed in the southern part of the island, from the coast inland to 600 meters. Older specimens were collected only after 1990, and most of the specimens were collected without food plant information. In one case, the insects were feeding on lavender, *Lavandula angustifolia* Miller (Lamiales: Lamiaceae). Several females of this sample were used to start a laboratory colony and were fed with *R. ulmifolius* .

**Figure 5.**
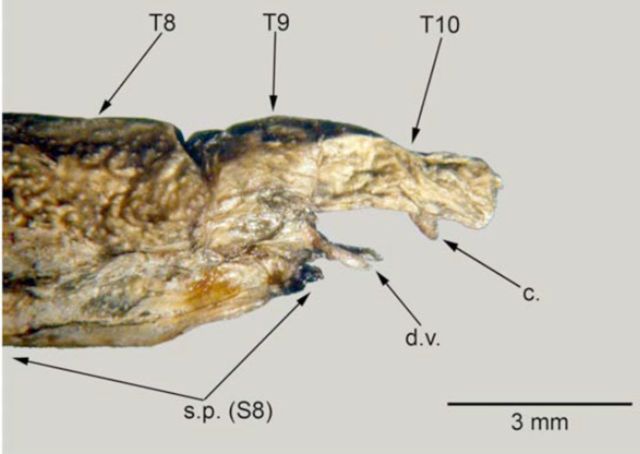
Last abdominal segments of a *Carausius morosus* abnormal female: T – tergites, S – sternite, c – cerci, d.v. – dorsal valvulae, s.p. – subgenital plate. High quality figures are available online.

**Doubtful specimen:** Among the females collected on *L. angustifolia* was a specimen with external characteristics of females but abnormal genitalia. This specimen, which was killed shortly after being collected, had a body with cuticle granulated and matt, similar to other females. The antennae were long, but not reaching 0.66 of body length as in the males. The meso and metathoraxic sternites did not show any reddish coloration as in males, and the general dimensions were those of a typical female. The external genitalia deformity of this specimen affected only the last three abdominal segments. Compared with normal females ( Figure 5 ), tergites 8 to 10 were still identifiable, although differently shaped. However, on the ventral side the subgenital plate (s.p.) was greatly reduced, not surpassing tergite 9 when it should have reached the apex of tergite 10. This undeveloped subgenital plate left exposed a pair of appendices, which under high magnification were revealed to be the dorsal valvulae (d.v.). The other ovipositor components, the inner and ventral valvulae, were much reduced, but discernible if viewed from a different angle. Also identifiable on this deformed female were the cerci (c.).

**Figure 6.**
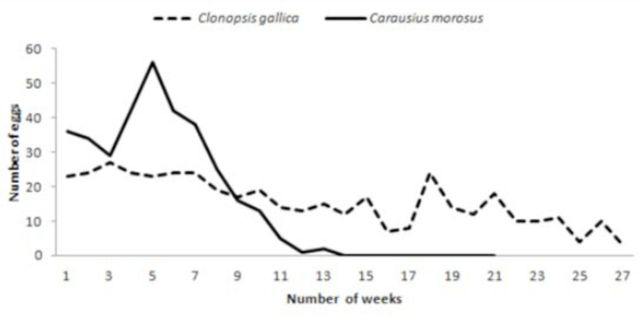
Number of eggs (y axis) laid by a female *C. morosus* and *C. gallica* weekly (x axis) during a 27-week period. High quality figures are available online.

**Figure 7.**
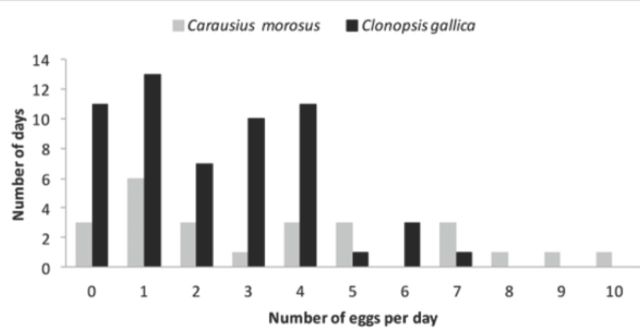
Number of days (y axis) in which were collected, from 0 to 11 eggs per day (x axis) for *Carausius morosus* and *Clonopsis gallica.* High quality figures are available online.

**Oviposition:** An adult female kept in laboratory from 28.vi.2010 to 26.xi.2010 laid 339 eggs. The comparatively large oviposition rate was similar to that observed by other authors ( Roth 1916 ). The number of eggs laid per day during the oviposition period observed over 25 days varied greatly between 0 and 10 (the mean number of eggs per day was 3.76). This was considerably larger than the mean number of eggs calculated for the whole oviposition period (2.26 eggs per day) ( Figure 7 ). The number of eggs laid decreased considerably after the 11th week with less than one egg produced per day. The postovipositional period lasted eight weeks ( Figure 6 ). According to Lelong (1995) , *C. morosus* only reproduces sexually in its Asian country of origin; in Europe, populations are made up exclusively of females. The males are reported to be very rare, and we were unable to observe any in the field or in the laboratory population.

Infraorder **Areolatae**

Family **Bacillidae**

***Clonopsis gallica*** ( Charpentier 1825 )

*Phasma gallicum* , Charpentier 1825 Horae entom, p.94. [Type locality: Europe, South Western Europe, France, Southern]

*Bacillus granulatus,* Brullé 1832 Exp. sc. de Morée Ins., p.84, t.29, Figure 6 .

*Bacillus gallicus* var. *occidentalis,*Bolívar 1894 An. Soc. esp. Hist. Nat., 23: 73

*Bacillus affinis,* Salfi 1925 , Ann. Mus. Zool. Uni. Napoli, 5(12):1

Material studied (13 females): MMF 33667 – 1F, Santo da Serra, 28SCB3121, 06.vii.1952; MMF 27693 – 1F, Monte, Funchal, 28SCB2216, 24.viii.1981, R. Garton leg.; MMF 27022 – 1F, Boa Nova, Funchal, 28SCB2314, viii.1996, Maria da Luz leg.; MMF 36294 – 1F, Caminho da Choupana nº173, 28SCB2215, 12.ix.2001, José Exequiel Rodrigues leg.; ICLAM 0992 – 1F, Confeiteira, Monte, 450 m, 28SCB2216, 16.viii.2002, António Domingos Abreu leg.; UMa – 1F, São Gonçalo, Funchal, 28SCB2313, 5.viii.2003, Rogério Correia leg.; ICLAM 03239 – 1F, Urbanização Portada de Santo António, Monte, Funchal, 28SCB2115, 409 m, 20.viii.2004, Rui V. Silva leg.; ICLAM 03240 – 1F, Urbanização Portada de Santo António, Monte, Funchal, 28SCB2115, 409 m, 1.vi.2005, Rui V. Silva leg.; MMF 36290 – 1F, Longueira, Campanário, 28SCB1016, 7.viii.2005, Ysabel Gonçalves leg.; UMa – 1F, Gaula, Santa Cruz, 28SCB2916, 22.vi.2006, Gilda Freitas leg.; UMa -1F, Camacha, Santa Cruz, 28SCB2716, 31.viii.2008, João Reis leg.; ICLAM 03238 – 1F, Assomada, Caniço, Santa Cruz, 28SCB2914, 15.iv.2010, Lina Noite leg.; MMF 41446 -1F, Longueira, Campanário, 28SCB1016, 20.vii.2010, Hilário Sousa leg.

*Clonopsis gallica* is a circum-Mediterranean species, which extends its distribution in mainland Europe and Africa throughout Portugal, Spain, France, Italy, Greece, and North Africa (excluding the Sinai Peninsula). It is also present in many Mediterranean Islands. Although this species went more or less unnoticed by entomologists in Madeira from many decades, first reports date back to 1952, and the seven specimens studied so far clearly suggest that this species is located in the southern parts of Madeira.

**Oviposition:** The single specimen of *C. gallica* kept in the laboratory showed a long preoviposition period of 11 weeks after which it suddenly started to oviposit. The oviposition period lasted 27 weeks (191 days), and during this period 426 eggs were laid. The number of eggs laid per day varied from zero to seven eggs with a mean number of 2.23 eggs per day ( Figure 7 ). During the ovipositional period, the number of eggs laid decreased after the 15th week and increased again in the 18th week ( Figure 6 ). This female continued to lay eggs until it died, but in the last three weeks the number of eggs laid was less than one per day. This species is an obligate parthenogen. Males were never observed in fieldwork or laboratory populations.

## Discussion

### Phasmids of Madeira

**Figure 8.**
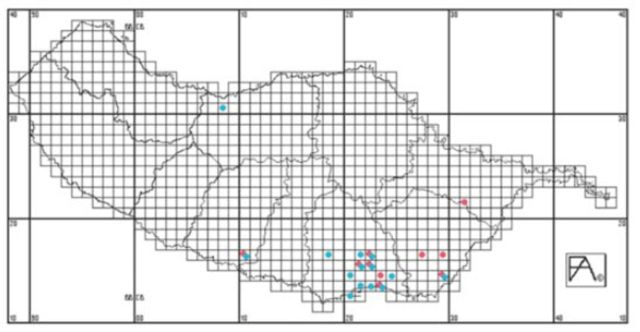
Distribution map with the collection localities plotted for *Carausius morosus* (blue dots) and *Clonopsis gallica* (red dots). The island is divided in 10 municipalities. The distribution map uses the UTM coordinate system (South East Base Datum), and each coordinate associated with a record identifies a specific 1 km square quadricule. High quality figures are available online.

A study of the specimens deposited in several institutional collections across Madeira revealed that the first collected specimen belongs to *C. gallica* and dates back to 1952. Since 2003, several specimens of this and *C. morosus* have been collected each year. *Carausius morosus* is an exotic species originally from Shembaganur, oriental India, and is widely-known in Europe as a laboratory insect for over a century, since it was imported by R. P. Pantel in 1897 for the laboratorial study of parthenogenesis. Due to its fecundity, long life span, and the relative ease with which it can be reared, it is a widely commercialized species. It is an attractive pet for collectors, and these stick insects are often kept in dense colonies for scientific use or for science education programs and demonstrations in schools. It has been introduced in Florida ( Denmark and Porter 1973 ), California ( Brock 1992 ), the U.K. ( Lee 1993 ), Germany ( Weidner 1981 ), South Africa ( Brock 1999 ), and Madagascar ( Cliquennois 2007 ). This species currently seems to be rather common in Madeira, but the first specimens were sampled only after 1990. Although it is not possible to know whether it has been recently introduced, the larger number of records in the last year suggests that this species is increasing its area of distribution on the island. In contrast, *C. gallica* , which has been present at least since 1952 seems to be more localised. The distribution and comparatively larger oviposition rate of *C. morosus* kept in laboratory suggests that in Madeira this species is probably expanding its area at a more rapid rate than *C. gallica.* Both species were mostly collected from southern coastal localities, predominantly in the Municipality of Funchal ( Figure 8 ), and no evidence is known to us that supports that they escaped or were introduced as stowaways from the port of Funchal, although this is not excluded especially for *C. morosus* .

The origin of phasmids in Macaronesian archipelagos is unknown, and although both species are considered to be introduced or possibly introduced ( Arechavaleta et al. 2005 ; Sousa 2010 ), a native origin for *C. gallica* cannot be excluded. The first reason is that *C. gallica* is present in Morocco, the closest mainland area to Madeira, 635 km to the east. According to recent studies on reproductive and chromosomal diversification, Morocco (the Rif region) is considered to be the radiation centre of the genus *Clonopsis* ( Scali and Milani 2009 ). In Morocco, the genus *Clonopsis* includes two other species in addition to *C. gallica* , *C. algerica* and *C. maroccana* , but the relationship between them is unknown ( Milani et al. 2009 ). A second reason is that Madeira is an oceanic island, and geological evidences show that it has never been connected to mainland; therefore, the only possible ways for phasmids to colonize Madeira are either by natural means or as stowaways. Although phasmids have a limited power of dispersal due to inactive and strictly herbivorous habits, they are in fact successful island colonizers. A proof of this capability is illustrated by the presence of phasmids in isolated archipelagos composed of oceanic islands. A great number of phasmid species are known to be present on the Pacific oceanic islands, with more than 60 species belonging to 30 genera ( Nakata 1961 ). Their considerable polyphagia and parthenogenetic mode of reproduction may help them to establish populations after reaching these new insular areas.

*Carausius morosus* and *Clonopsis gallica* are two obligate parthenoforms that occasionally produce males. According to Lelong (1995) , *C. morosus* has sexual reproduction only in its country of origin and reproduces by thelytokous parthenogenesis in other regions; here, males are very rare ( Pijnacker and Ferwerda 1980 ). In laboratory populations of the parthenogenetic *C. morosus* , both males and masculinised females occasionally appear. Masculinised females of spontaneous origin may be either intersexes and/or gynandromorphs (Pickjaker 1964). Gynandromorphs have genetically male and female tissues. Intersexes are genetically uniform. They may be intermediate between typical female or male genotypes, or purely male or female genotypes with some parts of the body with a phenotype opposite to their genetic sex (Narita et al. 2010). The abnormal female-like specimen collected on *L. angustifolia* is a significant find given that only 22 specimens of *C. morosus* were sampled. Because we did not examine the follicle cells, it is not possible to know the genetic sex of the specimen, but external genitalia correspond to a female, some parts of which were not fully developed. Identification of stick insects is difficult because body and karyotype ‘phenotypes’ are considerably independent ( Milani et al. 2009 ). Genetic diversity is related to their ability to overcome speciesspecific reproductive isolation mechanisms through hybridization, polyploidy, parthenogenesis, hybridogenesis, and androgenesis ( Milani et al. 2010 ). This gives rise to individuals with both male and female morphological and/or genetic characters (i.e., intersexes and gynandromorphs).

### Communicating biodiversity to public and the need for faunistics

Invertebrates are by far the largest group of all living beings and they are of enormous importance to general conservation because of their biomass and the ecological services they provide. Despite this, worldwide awareness of invertebrate conservation, particularly of insects, is a rare phenomenon, with the exception of a few species of groups such as butterflies. This lack of awareness not only affects invertebrates. According to recent Eurobarometer (2007) surveys, only 35% of the European population knows what biodiversity means. The term “biodiversity” itself is not clearly and generally understood, because it refers to a very complex concept. It has been suggested that awareness and experience of biodiversity at an early age are important for the future development of understanding ( Helldén and Helldén 2008 ). Feelings and beliefs about the environment determine people’s attitudes ( Pooley 2000 ). Negative attitudes among adults particularly towards insects and other less popular animals are common and can be highly resistant to change ( Bjerke et al. 2003 ). However, there are cases of success among insects as the tree lobsters, *Dryococelus australis* , a phasmid from Lord Howe Island that is considered the rarest insect of the world. This species, once thought to be extinct, was rediscovered in 2001 on a volcanic offshore islet off the cost of mainland Australia, producing an enormous interest among public and insect conservationists (Pridel et al. 2003).

The form of appreciating insects varies according to age and different cultures ( Vargas 2006 ). Children are ideal recipients for teaching about biodiversity and conservation because of their tremendous capacity for learning ( Matthews et al. 1997 ; Balmford et al. 2002 ). Communicating biodiversity to adults is more difficult. According to the Eurobarometer survey (2007), the most typical ways that Europeans learn about biodiversity issues are watching news and documentaries on TV, searching the Internet, and reading newspapers and magazines. Mass media are important tools for disseminating science. The collaboration of scientists with journalists can be valuable for selecting a news story based on what is important to the readers, not the scientists ( Bishop 1997 ). Faunistics is considered *soft science* and of little interest for publication by many researchers. During their career, biologists are under great pressure to focus on more conceptual articles and so see no reason to spend precious time and energy on faunistic publications. However, this information is not only a useful base for biodiversity conservation, but is also of interest to the general public and, as such, increases their awareness and interest in biodiversity conservation. Communicating biodiversity science issues in ways that are useful and meaningful for science and society remains a challenge. The solution may be in more effective communication and partnership with newspapers. This could be particularly useful in areas where resources for communicating with the public are limited.
